# Overcoming the Challenge of Singing Among Cochlear Implant Users: An Analysis of the Disrupted Feedback Loop and Strategies for Improvement

**DOI:** 10.3390/brainsci15111192

**Published:** 2025-11-04

**Authors:** Stephanie M. Younan, Emmeline Y. Lin, Brooke Barry, Arjun Kurup, Karen C. Barrett, Nicole T. Jiam

**Affiliations:** 1 Department of Head and Neck Surgery, University of California, San Francisco, CA 94143, USA; stephanie.younan@ucsf.edu (S.M.Y.); emmeline_lin@hms.harvard.edu (E.Y.L.); brooke.barry@ucsf.edu (B.B.); 1069206@lammersvilleusd.net (A.K.); karen.barrett@ucsf.edu (K.C.B.); 2 Institute for Health and Aging, University of California, San Francisco, CA 94143, USA

**Keywords:** cochlear implant, singing, vocal production, music perception, auditory–motor feedback loop, pitch perception, prosody, aural rehabilitation, neuroplasticity

## Abstract

**Background:** Cochlear implants (CIs) are transformative neuroprosthetics that restore speech perception for individuals with severe-to-profound hearing loss. However, temporal envelope cues are well-represented within the signal processing, while spectral envelope cues are poorly accessed by CI users, resulting in substantial deficits compared to normal-hearing individuals. This profoundly impairs the perception of complex auditory stimuli like music and vocal prosody, significantly impacting users’ quality of life, social engagement, and artistic expression. **Methods:** This narrative review synthesizes research on CI signal-processing limitations, perceptual and production challenges in music and singing, the role of the auditory–motor feedback loop, and strategies for improvement, including rehabilitation, technology, and the influence of neuroplasticity and sensitive developmental periods. **Results:** The degraded signal causes marked deficits in pitch, timbre, and vocal emotion perception. Critically, this impoverished input functionally breaks the high-fidelity auditory–motor feedback loop essential for vocal control, transforming it from a precise fine-tuner into a gross error detector sensitive only to massive pitch shifts (~6 semitones). This neurophysiological breakdown directly causes pervasive pitch inaccuracies and melodic distortion in singing. Despite these challenges, improvements are possible through advanced sound-processing strategies, targeted auditory–motor training that leverages neuroplasticity, and capitalizing on sensitive periods for auditory development. **Conclusions:** The standard CI signal creates a fundamental neurophysiological barrier to singing. Overcoming this requires a paradigm shift toward holistic, patient-centered care that moves beyond speech-centric goals. Integrating personalized, music-based rehabilitation with advanced CI programming is essential for improving vocal production, fostering musical engagement, and ultimately enhancing the overall quality of life for CI users.

## 1. Introduction

### 1.1. Cochlear Implantation: Triumphs and Tribulations

The cochlear implant (CI) stands as one of the most transformative neuroprosthetic devices of the modern era, offering a profound restoration of sound perception for individuals who meet the rigorous candidacy criteria [1]. While historically defined by severe-to-profound hearing loss, these criteria now more accurately reflect individuals who derive limited benefit from conventional hearing aids [2]. The technology has advanced rapidly from its early days, when expectations were generally modest and the primary goal was to provide an awareness of environmental sounds and cues for lip-reading [1]. Early single-electrode devices conveyed basic sound sensations, a significant achievement at the time, but fell short of realistically representing complex sounds—a challenge that persists with modern technology.

Today, with the advent of multichannel technology and sophisticated sound processing, most recipients can achieve high levels of open-set speech recognition in quiet listening conditions, enabling spoken communication without reliance on visual cues [1]. This success has redefined the possibilities for individuals who would otherwise live in a world of limited sound and has cemented the CI’s role as a standard of care for profound hearing loss.

However, this remarkable triumph in restoring speech is shadowed by a persistent and significant challenge: the ability to perceive complex, non-speech auditory signals. Specifically, CI users consistently report profound difficulties with both the perception of music and the nuanced paralinguistic information conveyed through the human voice, such as emotional tone and linguistic prosody [3,4]. The auditory signal in these settings is often described as “spectrally impoverished” or “spectrally coarse”, lacking the detailed frequency and timing information necessary to resolve music- and emotion-specific cues based on pitch and vocal inflections [4,5]. This impaired musical sound perception is a central challenge that impacts quality of life, emotional communication, social involvement, and the ability to engage in artistic and social activities [6,7]. Furthermore, recent systematic reviews confirm that while hearing loss and hearing devices have a substantial negative impact on music perception, the crucial psychosocial dimensions of music appreciation, enjoyment, and social participation have historically been overlooked in research focused solely on acoustic metrics [8].

Research has revealed a “perception-appraisal gap”, a concept also accurately described as diminished music appreciation, where objective perceptual accuracy on music tasks (e.g., melody recognition) does not consistently correlate with subjective enjoyment or appraisal ratings [9]. Leading work by Looi, Gfeller, and colleagues emphasizes that music appreciation and enjoyment must be considered valid clinical outcomes, as they represent a complex, holistic experience shaped by factors such as memory, social context, and rhythm, transcending mere acoustic discrimination [10]. This gap illustrates that the primary motivation for engaging with complex auditory stimuli is experiential and emotional, not merely acoustic accuracy. This experiential necessity is quantifiable. For instance, a recent study found that vocal emotion recognition performance (the ability to correctly interpret the emotional nuances in a speaker’s voice) is a more significant predictor of self-reported Quality of Life (QoL) in adult CI users than are traditional clinical measures of sentence recognition [9]. Because vocal prosody and emotion function as the essential ‘musical components of speech’, and because their successful perception is so critical to social connection and QoL, this evidence provides a powerful rationale for elevating the study of music and voice perception from a secondary hearing concern to a primary goal in CI research and rehabilitation. This clinical imperative sets the stage for our subsequent analysis of singing, which requires the highest degree of precise control over these same fundamental spectral and prosodic cues.

### 1.2. Structure of the Review

This narrative review synthesizes the current knowledge focusing on the mechanistic basis of pitch-dependent perceptual and production challenges in music and singing. The initial sections detail the fundamental perceptual challenges CI users face with music and vocal prosody, which stem in part from the limitations in perceiving the electric signal delivered to the cochlear neurons, and examine the consequent difficulties in vocal production, particularly singing, which are inextricably linked to perception via the auditory–motor feedback loop. Subsequently, the review explores the primary pathways to improvement by analyzing technological advancements in sound processing, the efficacy of targeted rehabilitative strategies, and the critical role of neuroplasticity and individual patient factors, with a particular focus on the evidence for sensitive periods in auditory development. These findings are then integrated into a cohesive model that examines the mechanistic basis for production deficits, the complex relationship between perception, production, and enjoyment, and the need for a more holistic approach to auditory rehabilitation. The review concludes by summarizing the key findings and proposing a forward-looking agenda for research and clinical practice aimed at moving beyond speech intelligibility to restore the full spectrum of human auditory experience.

## 2. Methods

A narrative review was conducted using PubMed, Web of Science, and Embase to source articles on the following topics: (1) limitations in CI signal processing and technology; (2) challenges in music and vocal perception in CI users; (3) the breakdown of the auditory–motor feedback loop during complex vocal perception tasks such as singing; (4) strategies to overcoming the perceptual challenges of CI, including auditory rehabilitation strategies such as music-based training; and (5) neuroplasticity and sensitive periods in the context of singing and hearing restoration. Search terms included “cochlear implant”, “cochlear implantation”, “singing”, “vocal production”, “music perception”, “prosody”, “emotional prosody”, “auditory training”, “aural rehabilitation”, and “neuroplasticity”. Articles were reviewed by three authors (SMY, EYL, BB) for their relevance to each topic, and their reference lists were further hand-searched to include any other relevant studies. The search was conducted in August 2025, without formal exclusion criteria based on publication date. Articles that were considered irrelevant, published in a language other than English, and/or consisted of only an abstract were excluded.

## 3. The Challenge of Auditory Perception Through an Impoverished Electric Signal

**Figure 1:**
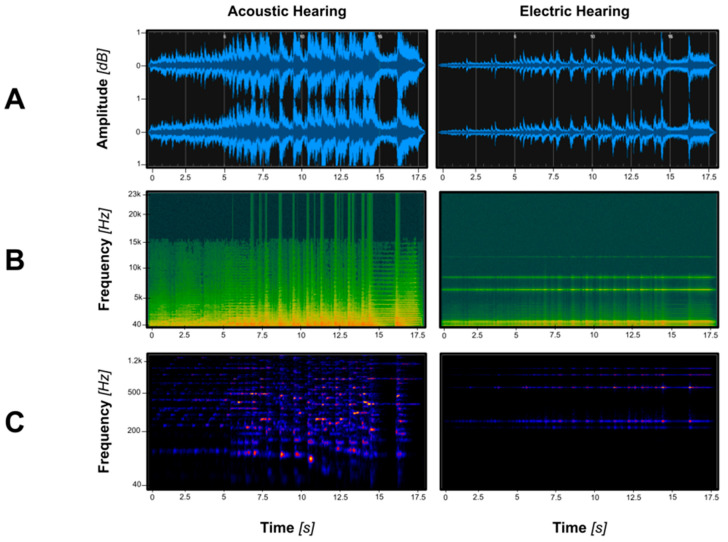
Cochlear Implant (CI) vs. Normal Hearing (NH) Spectrography and Waveforms. All figures were created with an 18 s clip of Gershwin’s “Rhapsody in Blue”. CI audio simulation created through a vocoder chain utilizing frequency assignment to pure tones. (**A**) displays the acoustic (NH) waveform on the left-hand side of the diagram in comparison to the right-hand electric (CI) waveform. (**B**) displays both electric and acoustic spectrograms, with frequencies ranging from 40 Hz to 23 kHz. (**C**) is a spectrogram limited to a range of 40 Hz to 1.2 kHz, focusing on the traditional melodic range and emphasizing the loss of melodic elements in CI hearing.

Despite the technological advancement of CIs in restoring access to speech and communication for individuals with hearing loss, the electric signal delivered by a CI is fundamentally different from the full, acoustic signal experienced through typical hearing. While CIs are adequate for transmitting most of the linguistic information essential for communication, the acoustic features that compose music and prosody perception appear more degraded to listeners. While individual variability in performance exists and highlights the role that individual factors may play in complex sound perception, technological limitations within the device must also be considered. CIs encode sound by dividing incoming acoustic information into a limited number of frequency channels (typically 12 to 22, depending on the manufacturer and model); the envelope of each channel is then converted to electrical pulses that are delivered to specific electrodes in the intracochlear array [1,11,12]. Each electrode stimulates a specific region along the basilar membrane of the cochlea that corresponds with a specific frequency range (and therefore pitch), following the natural tonotopic organization of the cochlea [1]. Current, conventional CI sound coding strategies, such as Continuous Interleaved Sampling (CIS) Advanced Combination Encoder (ACE), primarily extract and transmit the temporal envelope of sound, which consists of the slow-varying contours in amplitude (i.e., loudness) over time [1,13]. These envelope cues are concentrated within the 2–50 Hz range and are essential for identifying speech phonemes and achieving basic speech recognition, especially in quiet conditions [7,14,15]. Higher envelope frequencies (such as 200 Hz) may also be present in the electrical signal, though they are not reliably encoded or perceived by all CI users [14,16]. Ultimately, while temporal envelope cues are well-represented by CI signal processing, two other critical components of sound, spectral resolution and temporal fine structure, are not [1,7]. This limitation results in the severe degradation and simplification of a sound waveform through a CI, which can be visualized in Figure 1.

Spectral resolution describes the ability to distinguish different, but closely spaced, frequencies within a source of sound. In a typical hearing individual, this precision is enabled by the thousands of hair cells within the cochlea detecting sound wave vibrations [17]. In contrast, the transmission of fine spectral details is significantly impaired in CI users, leading to a loss of melodic elements in electric hearing (Figure 1). This simplification is due to the small number of electrode channels, place–pitch mismatch (the misalignment of the CI electrode contacts with the tonotopic map of the cochlea, so that the frequencies assigned to the electrodes do not correspond to the characteristic frequencies of the spiral ganglion neurons at those locations), wide bandpass filters, and broad current spread of a CI [5,18,19,20]. The latter contributes to channel interaction, which occurs when the electrical current from one electrode spreads and stimulates adjacent electrodes, leading to overlapping neural excitation and reduced clarity of sound [21]. This results in diminished spectral resolution and increased spectral smearing (i.e., the blurring of different frequency components within the acoustic signal), which has a variety of consequences, such as difficulty in understanding speech in noise or other complex auditory environments, sound localization, and pitch and timbre perception [5,20,22].

Temporal fine structure (TFS) refers to the rapid fluctuations (from 600 Hz to 10,000 Hz) within each cycle of an acoustic waveform, which encode information about the phase and timing of sound waves [23]. Similarly to the information provided by spectral resolution, the subtle cues of TFS are crucial for accurate, fine-grained pitch perception, speech understanding in noise, and other complex listening tasks that require transmission of precise signals beyond what is encapsulated by the temporal envelope of sound [23,24]. Specifically, the perceptual deficits of the lost fine structure cues are noticeable and exacerbated in noisy auditory environments, where the ability to segregate and attend to relevant voices or sounds relies heavily on both subtle spectral and temporal cues [25,26]. This can make group conversations, crowded events, or musical performances particularly challenging for CI users. These fundamental limitations in transmitting spectral and temporal information lead to a cascade of perceptual challenges across different auditory domains, as summarized in Table 1.

### 3.1. CI Deficits When Applied to Music Perception

The limited transmission of spectro-temporal fine structure information contributes to the impoverished signal of a CI, leading to a flattened or inaccurate auditory experience that makes music difficult to properly hear and/or enjoy. In fact, the inability to fully appreciate music is one of the most common complaints among post-lingually deafened CI users. For some individuals, music is perceived as discordant, unpleasant, or simply as noise, leading to avoidance of musical activities that were once a source of joy and social connection [6,24]. As described in Table 1, one of the most significant limitations for CI users is pitch perception, which underlies multiple other components of music including identifying melodies, harmonics, polyphonic sound, and more. Standardized tools such as the Montreal Battery of Evaluation of Amusia (MBEA) or the Clinical Assessment of Music Perception (CAMP) consistently demonstrate impaired performance in many of these dimensions among CI users as compared to normal hearing listeners [27,28].

Beyond the aforementioned limitations in spectral discrimination and transmission of TFS, the marked challenges of accurate pitch perception faced by CI users can be attributed to a variety of reasons. For example, CIs may compress the frequency of the sound input, reducing the changes in pitch between adjacent electrodes in comparison to what a normal-hearing individual would perceive within the same regions along the basilar membrane [29]. This frequency compression can lead to CI users needing larger pitch intervals to distinguish changes in sound [3,30]; studies have found that pitch discrimination thresholds in adult CI users are around 3 semitones, whereas normal-hearing listeners can identify differences of less than 1 semitone [18,29]. While this difference may seem minor, changes of 1–3 semitones have a significant impact on music perception. Additionally, some electrodes are positioned more basally in the cochlea than the location predicted by place–pitch mapping functions due to the limited length of the electrode array and anatomical constraints of patients. This mismatch leads to acoustic pitches being electrically perceived as different frequencies than reality [31,32,33]. However, some studies have shown that CI users may eventually be able to adjust and adapt to the mismatch over months to years of device use, indicating that the perception of reality can change due to neural plasticity [34,35,36]. The missing electrical stimulation in the most apical (deep) regions of the cochlea may also limit the low-frequency sounds heard by CI users. While longer electrode arrays may help minimize the magnitude of this mismatch, this issue persists as CI arrays often do not reach the cochlear apex [31,32]. This discrepancy serves as one of the many contributors to CI users performing worse than normal hearing listeners on both pitch identification and discrimination tasks [22].

The impacts of inaccurate pitch perception are multifold. CI users possess impaired ability to recognize melodic contour, or the changes in pitch across a sequence of musical notes that make up the distinct melody of a musical piece [37]. Moreover, CIs limit accurate encoding of the harmonic relationships that underlie consonance and dissonance, and CI users may be unable to reliably distinguish between consonant (pleasant) and dissonant (unpleasant) intervals or chords [38,39]. Furthermore, CI users struggle with tasks that require the integration of multiple simultaneous pitches, known as polyphonic sound, perceiving them as fused instead of distinct [40]. All of these challenges may be associated with a blunted ability to perceive the nuanced and complex dimensions of music, thereby impacting the subjective level of enjoyment experienced by, as well as the emotional response evoked within, the listener. Studies assessing the change in emotions such as happiness and sadness (measured as valence), or excitement and fear (measured as arousal), after listening to music have all been found to be decreased among CI users as compared to normal-hearing individuals [41,42].

CI users also experience significant difficulty with timbre discrimination, or the ability to hear the tone qualities that differentiate two instruments playing the same note (more specifically, the same pitch and amplitude) [24,43]. As a result, CI users tend to confuse instruments across instrumental families [44]. This impacts their ability to listen to music in the real-world, as many musical compositions consist of multiple instruments playing sequentially or simultaneously. Finally, a third essential component to music perception is rhythm, which consists of music’s tempo and meter, and is transmitted through the temporal envelope of sound. In contrast to pitch and timbre, basic rhythm perception is relatively preserved in CI users [45]. In fact, when processing the emotion in auditory cues, CI users may compensate for the missing pitch perception through an increased reliance on tempo cues [46,47].

### 3.2. Music-Related CI Deficits as Applied to Vocal Perception

Beyond music, the spectrally degraded CI signal affects the perception of complex vocal qualities such as speech prosody cues and voice emotion, which are often conveyed by subtle but salient changes in the speaker’s pitch, tone, and rhythm [48,49]. Vocal prosody, which can be thought of as the musical components of speech, encompasses the intent and affect (emotion), contrastive stress patterns (emphasis placed on certain words), intonation patterns (i.e., pitch contour, with rising pitch indicating continuation while falling pitch suggesting termination), intensity, and duration of speech. These elements are all essential for daily, interpersonal interactions by conveying meaning beyond words [48,50]. Many aspects of human language are reliant on accurate detection of prosody, including identifying pragmatic intent (e.g., distinguishing a question from a statement) and recognizing sarcasm and emotion (such as happy, sad, angry, scared, or relieved) [48]. Because the processing constraints of a CI device can degrade prosodic cues, CI users have consistently demonstrated impaired performance on voice emotion recognition tasks compared to normal-hearing individuals [41,51,52]. Such assessments incorporated stimuli such as neutral statements, nonce words (which are sequences of letters or sounds that form a “word” not part of the English language), or CI-simulated sounds delivered with a specific emotion, and found that CI users were significantly worse at correctly recognizing the targeted emotion [49,53,54]. These incomplete signals can lead to frequent misinterpretation or flattened affect in social communication, creating a sense of social disconnection or isolation [41,52]. For pediatric CI users, speaking with an exaggerated prosody (i.e., in a child-directed manner, which features greater variability in pitch and intensity) has been found to help this population more accurately perceive and understand vocal emotion [55], but consistent implementation of this technique may be difficult to achieve in the real world and across age groups. Ultimately, these documented challenges with complex vocal perception may persist as or translate into certain dimensions of vocal production that require intentional changes in pitch as related to emotion and intonation, such as singing.

## 4. The Challenge of Production: The Broken Feedback Loop in Singing

The act of singing, which demands a far higher degree of pitch precision than conversational speech, exposes the critical limitations of the auditory information provided by the CI. Specifically, the spectrally degraded signal functionally hinders the high-fidelity auditory–motor feedback loop, a sensorimotor circuit essential for the real-time monitoring and correction of vocal output. This section will deconstruct this neurophysiological breakdown, beginning with an explanation of the feedback loop’s function in normal hearing, followed by an analysis of how the CI signal compromises this mechanism, and culminating in a detailed review of the acoustic evidence that quantifies the profound and multifaceted deficits in the singing production of CI users.

### 4.1. The Auditory–Motor Feedback Loop: A Foundation for Vocal Control

The production of any complex, learned vocalization, from speech to song, is governed by a sensorimotor control system known as the auditory–motor feedback loop. This neural circuit allows for the continuous, dynamic regulation of vocal output based on sensory input. The process begins in the motor cortex, which generates not only the primary (motor) command sent to the laryngeal and respiratory muscles to produce sound, but also a simultaneous corollary discharge, or “efference copy”, to the associated sensory areas (i.e., auditory cortex) to inform them of the impending motor actions [56]. In other words, this efference copy serves as a sensory prediction (an internal simulation of the expected auditory outcome of the motor command) which helps the individual anticipate the sound of their own voice before it actually arrives to their ears [57,58].

In a parallel stream, the actual sound produced is captured by the auditory system and transmitted as afferent sensory feedback to the auditory cortex. Here, a critical comparison occurs: the brain matches the incoming, real-world auditory feedback against the internally generated prediction [57,58]. If the two signals match, the motor command is deemed correct. However, if a discrepancy (or error signal) is detected, the system initiates a rapid, often subconscious, compensatory adjustment to the vocal motor output to minimize the error. This continuous process of prediction, comparison, and correction is fundamental to both the acquisition of speech in infancy, which occurs during a critical developmental window, and the moment-to-moment maintenance of accuracy and stability in mature vocalizations [56,57].

The precision of this system in individuals with normal hearing is remarkable. Its sensitivity can be quantified using vocal perturbation paradigms, where a participant’s real-time auditory feedback is experimentally altered without their knowledge. When the pitch of the feedback is artificially shifted, speakers reflexively and rapidly compensate by adjusting their vocal pitch in the opposite direction of the perceived shift. The threshold for initiating this compensatory response reveals the system’s sensitivity. While vocally untrained individuals begin to correct for pitch shifts of approximately a quarter of a semitone, the auditory–motor loop in trained singers is significantly more refined [56]. Their extensive vocal practice enhances the neural plasticity of this circuit, lowering their response threshold to shifts as small as 0.06 of a semitone [56]. This demonstrates that the feedback system is not a static, hard-wired mechanism but a dynamic, experience-dependent circuit. The very act of singing trains the brain to become more adept at detecting and correcting the minute pitch errors that are imperceptible in speech but musically salient. Consequently, the standard for proficient singing is predicated on a neural feedback system capable of operating with a precision far finer than a single semitone.

### 4.2. The Impact of Degraded Auditory Feedback in Cochlear Implant Users

**Figure 2:**
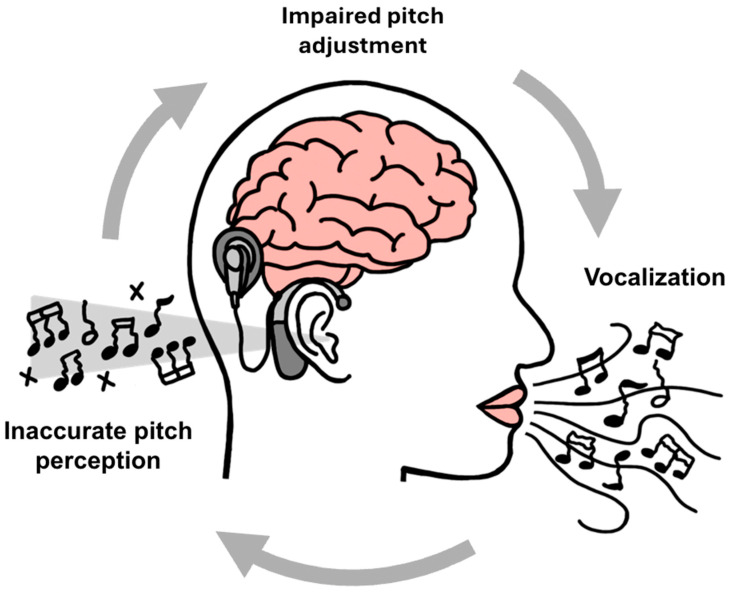
The broken auditory–motor feedback loop when individuals who use cochlear implants (CI) try to sing. Sound produced by the lips (vocalization) travels to the implanted ear(s) with profound hearing loss, but the technological limitations of the CI (device and/or sound processing) lead to inaccurate perception of pitch. In individuals with normal-hearing ears, the brain is able to compare the produced sound to the intended sound and make immediate adjustments to pitch as needed, correcting the intonation of the notes sung next. However, because CI users cannot accurately hear musical sound (afferent or sensory signal), they have impaired pitch adjustment that leads to a cycle of out-of-tune singing (efferent or motor response).

For a CI user, the auditory–motor feedback loop is not absent but rather compromised at its most critical juncture: the afferent sensory signal, as illustrated in Figure 2. As detailed in the preceding sections of this review, the electrical signal delivered by a CI is spectrally impoverished and smeared, lacking the fine-grained frequency and timing information that is essential for accurate pitch perception and resolving small differences in frequency [3].

When this degraded signal is fed back into the auditory–motor loop, its coarseness has a devastating effect on the error-detection mechanism. Small, musically relevant deviations in vocal pitch (for instance, singing half a semitone sharp) are not accurately perceived by the CI user. This failure occurs because the degraded signal results from miscommunication at the electrode-to-nerve interface, compounded by underdeveloped central processing and a damaged auditory system. From the brain’s perspective, the afferent feedback signal appears unchanged. As a result, the comparison between the predicted sensory outcome and the actual sensory feedback yields no mismatch, and thus, no error signal is generated. The system, therefore, remains “blind” to the production error, and no corrective motor adjustment is made, creating the cycle of out-of-tune singing as shown in Figure 2.

The magnitude of this breakdown has been starkly quantified in vocal perturbation studies conducted with CI users, typically postlingually deafened adults. In contrast to normal-hearing singers who respond to pitch shifts of 6 hundredths of a semitone, research has shown that adult CI users only initiate a compensatory vocal response when the pitch of their auditory feedback is shifted by a massive six semitones, or half an octave, a substantial error in vocalization [59]. This finding is paramount, as it provides definitive neurophysiological evidence that the auditory–motor system in CI users has undergone a fundamental functional transformation. It no longer operates as a high-precision, analog fine-tuning mechanism for maintaining pitch stability. Instead, it functions as a rudimentary, digital gross error detector.

This functional shift has important implications. The system’s primary role in normal-hearing vocalization is the continuous, subconscious stabilization of pitch around a target, which is near, if not fully, impossible among CI users. The feedback loop is only sensitive enough to intervene in the case of gross pitch deviations, on the scale of several semitones. Any pitch deviation smaller than the 6-semitone threshold deviation falls below the system’s threshold of detection and goes uncorrected. This renders the auditory–motor loop neurologically incapable of performing the primary task required for singing in-tune: maintaining a stable and accurate pitch reference. This functional mismatch explains why simple repetition and practice, without targeted perceptual training to improve the brain’s interpretation of the impoverished signal, often fail to yield significant improvements in the pitch accuracy of CI users’ singing [60].

It is critical to note, however, that the magnitude of this pitch deficit is highly variable and non-universal, influenced by multiple interacting factors. Biographical factors, such as the age of onset of deafness (pre- vs. post-lingual), and audiometric factors, including the extent of residual acoustic hearing and duration of device use, contribute significantly to this variability [18,61,62,63]. Furthermore, device-specific factors are paramount, as the precision of the pitch percept is directly affected by the electrode positioning, including the angular insertion depth, scalar location, and the electrode-to-modiolus distance [29,64].

### 4.3. Consequences for Vocal Production: An Acoustic Analysis of Singing Deficits

The direct consequences of this broken feedback loop have been observed and measured in the vocal output of CI users. Numerous studies have conducted detailed acoustic analyses of singing, comparing the pitch, timing, and quality of CI users’ productions to both target melodies and the performance of normal-hearing peers. This body of research provides compelling empirical evidence of a consistent pattern of deficits that can be traced directly back to the degraded auditory feedback.

#### 4.3.1. Pervasive Pitch Inaccuracy and Melodic Distortion

The most consistent and pronounced finding from acoustic analyses of CI users’ singing is a deficit in pitch control, which manifests across several key domains [60,61,65]. CI users struggle to reproduce even the most basic directional shape of a melody (i.e., melodic contour), a deficit that is particularly pronounced in those with prelingual deafness. Studies focusing on this population consistently report that their accuracy in producing the correct melodic contour—specifically a series of rising or falling notes—is near chance levels [60,65]. For example, one seminal study found a mean contour accuracy of just 52.3% in prelingually deafened CI users, where 50% represents random guessing [60]. While postlingually deafened adults may retain more accurate contour production, significant difficulties with overall pitch production remain a common challenge across CI user groups [66]. This indicates a fundamental difficulty in translating a perceived melodic shape into a corresponding series of vocal motor commands [67]. Furthermore, the pitch accuracy of individual sung notes is poor. The average deviation of a sung note from its intended target is consistently measured in the range of 2.3 to 2.7 semitones (230 to 270 cents), a level of error that is grossly out of tune and stands in stark contrast to the mean deviations of approximately 1.2 to 1.6 semitones observed in normal-hearing control groups [60,61,65]. The deviation that CI users demonstrated (2.3 to 2.7 semitones) reflects prior studies that found CI users are unable to distinguish two notes that are less than 3 semitones apart [18,29]. Finally, the singing of CI users is typically characterized by a severely compressed pitch range. Acoustic analyses reveal that the total span of pitches produced is often less than half of the range required by the target song, contributing to a vocal affect often perceived by listeners as “flat”, “monotone”, or lacking in melodic expression [60,65].

In stark contrast to these pervasive pitch-related deficits, the rhythmic accuracy of CI users’ singing is relatively strong. Because the temporal envelope of sound is well-preserved by standard CI processing strategies, users are often able to reproduce the timing and duration of notes with an accuracy comparable to that of their normal-hearing peers [65,68]. This divergence between poor pitch production and preserved rhythm production is a critical finding. It serves as a powerful internal control, demonstrating that the singing deficit is not a generalized problem with musical memory, motor planning, or the physical execution of a complex motor task. Instead, it isolates the problem to the specific domain of pitch-related feedback information; the one component of singing that is uniquely dependent on the fine-grained spectral and temporal fine structure information that the CI signal fails to provide.

#### 4.3.2. Loss of Tonal Center: The Inability to Maintain a Musical Key

The note-by-note inaccuracies described above have a cumulative effect that disrupts the entire tonal structure of a song. Western music is a relational system, where individual notes derive their musical meaning from their relationship to a stable tonal center, or key. Maintaining this key while singing requires the vocalist to make constant, small corrections to the intervals between successive notes, ensuring that each new note is produced in correct relation to both the preceding note and the overarching tonality.

For the CI user, this process breaks down. As established by vocal perturbation data, the auditory feedback loop is insensitive to the errors of several hundred cents that are typical in their singing [59,65]. Consequently, when a CI user sings a note that is, for example, 200 cents flat (i.e., lower than the intended target pitch), this error goes unperceived and uncorrected. The subsequent motor command for the next note is then planned relative to the previously sung (and incorrect) pitch, rather than being anchored to a stable, internal representation of the song’s key. This initiates a process of cumulative drift (Figure 2). Each uncorrected error provides a faulty reference for the next note, causing the singer to wander progressively and randomly further from the original tonal center. The result is a performance that is not merely out of tune on a note-by-note basis, but one that lacks a coherent and stable musical key, fundamentally undermining the melodic and harmonic structure of the song.

#### 4.3.3. Diminished Control over Vocal Quality

Beyond the primary issue of pitch accuracy, the breakdown of the auditory feedback loop also leads to diminished control over other musically relevant aspects of vocal quality. To understand these effects, it is useful to distinguish between clinical acoustic metrics of vocal pathology (e.g., jitter and shimmer) and metrics that reflect musical and expressive control (e.g., pitch stability and dynamic regulation).

The literature on clinical voice quality metrics in CI users presents a somewhat inconsistent picture. Jitter (cycle-to-cycle variations in frequency) and shimmer (cycle-to-cycle variations in amplitude) are often used to quantify hoarseness or vocal instability. Some acoustic analysis studies have found no significant differences in jitter and shimmer values between CI users and their normal-hearing peers, suggesting that the basic mechanics of phonation are not necessarily pathological [69,70]. In contrast, other studies have reported significantly more deviant jitter and shimmer in the CI population [71]. This discrepancy may arise because these micro-perturbations are heavily influenced by the baseline physiological state of the laryngeal system and may be less dependent on real-time auditory feedback compared to more deliberate vocal modulations.

A more consistent and telling pattern emerges when examining acoustic parameters that reflect higher-level musical control. Compared to normal-hearing individuals, CI users consistently demonstrate a higher mean fundamental frequency (F_0_), meaning they tend to speak and sing at a higher average pitch [69]. Their pitch is also less stable, showing greater F_0_ variability, which is a significantly larger standard deviation around the mean F_0_ over the course of a sustained vowel or phrase [60,61,65]. Additionally, they tend to have higher vocal intensity, or greater loudness [69]. This constellation of findings should not be interpreted as evidence of vocal pathology in the clinical sense (e.g., hoarseness), but rather as direct evidence of poor vocal regulation. Without the ability to precisely monitor their vocal output, CI users exhibit reduced control over the laryngeal muscle tension and subglottic pressure required to set and maintain a stable pitch and loudness target. This suggests a hierarchy of vocal control, where the most basic, reflexive stability of vocal fold vibration (measured by jitter and shimmer) may be less reliant on auditory feedback than the higher-order, intentional modulation of pitch, loudness, and timbre required for expressive speech and, especially, for singing. The failure of the CI feedback loop occurs primarily at this higher level of expressive control, reframing the “vocal quality” issue from one of potential pathology to one of diminished artistic and communicative capability. The various quantitative and neurophysiological findings detailed throughout the review, which demonstrate the profound mechanistic and behavioral deficits in pitch perception and production among CI users, are summarized in Table 2.

## 5. Pathways to Improvement

**Figure 3:**
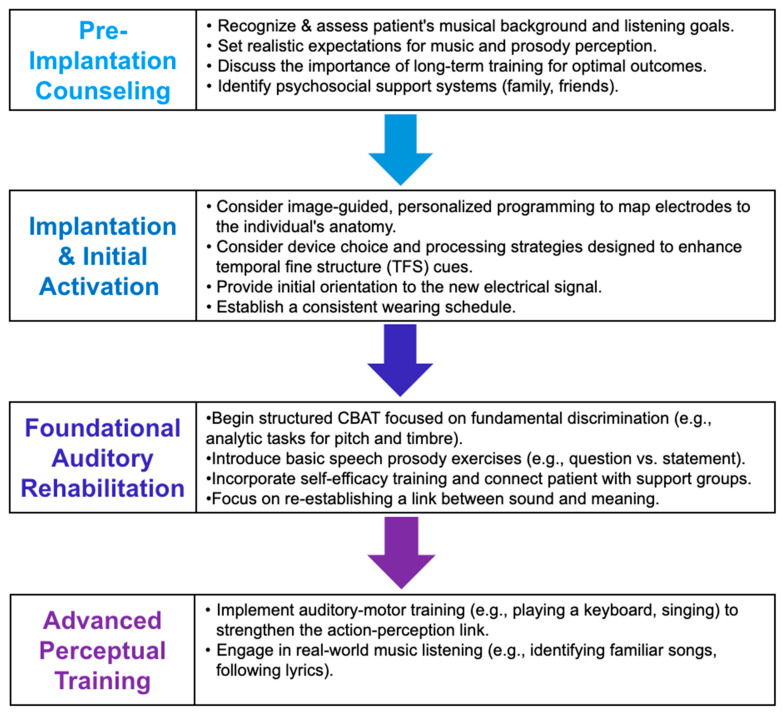
A Holistic Rehabilitation Plan for Music and Prosody Perception in Cochlear Implant (CI) Users. CBAT = computer-based auditory training.

While the challenges in music and voice perception for CI users are profound, they are not insurmountable. A growing body of research is dedicated to overcoming the limitations of the electric signal through a multi-pronged approach that combines technological innovation, targeted rehabilitation, and a deeper understanding of the brain’s capacity for adaptation. Figure 3 provides a holistic, step-by-step framework that summarizes strategies in each of these areas. These pathways offer promising avenues for improving not only perceptual accuracy but also the overall quality of the auditory experience for CI users. Crucially, the effectiveness of these pathways is often modulated by developmental factors, particularly whether auditory input is restored during the sensitive period for maximal neural plasticity.

### 5.1. Innovations in Sound-Processing Strategies

At the forefront of efforts to improve music perception is the development of more sophisticated sound processing strategies that aim to deliver a more detailed signal to the auditory nerve. These innovations focus on enhancing the two key areas degraded by conventional processing: spectral information and temporal fine structure.

#### 5.1.1. Enhancing Spectral Information

A primary limitation of CIs is their coarse spectral resolution, which is constrained by the finite number of physical electrodes. Two key strategies are being explored to address this. First, current steering aims to create “virtual channels” of stimulation. By delivering current simultaneously to two adjacent electrodes, the locus of maximal stimulation can be steered to points between the physical contacts, theoretically increasing the number of discriminable pitch percepts [12]. Strategies developed by Advanced Bionics, such as SpecRes (Spectral Resolution) and SineEx (Sinusoid Extraction), are based on this principle [74]. SpecRes uses a Fast Fourier Transform (FFT) and spectral peak-picking to deliver information with higher spectral resolution, while SineEx employs a psychoacoustic model to select and represent only the most perceptually important sinusoids in the signal [74]. While these strategies hold theoretical promise, clinical trials have shown considerable inter-subject variability, with mean performance often being similar to standard strategies, indicating the complex challenge of translating technological potential into consistent perceptual benefit [74].

Second, personalized programming represents a shift toward tailoring the device to the individual’s unique anatomy. Using post-operative CT scans, clinicians can create customized frequency maps that account for the precise location of each electrode within the cochlea [75]. This approach has yielded a crucial finding: the optimal electrode-to-nerve interface may differ for speech and music. Research from Galvin et al. (2007) [76] suggests that while closer proximity of electrodes to the modiolar wall (the nerve-rich core of the cochlea) improves speech perception, better music perception is associated with electrodes positioned farther away [76]. This may be because the broader electrical field from a more distant electrode, while less precise, stimulates a larger population of neurons. This wider activation could be more effective for representing the complex harmonic relationships inherent in music, whereas speech perception may benefit more from the highly localized stimulation provided by closer electrodes [76].

Clinicians can also create custom CI programs with certain electrodes deactivated, dependent on the amount of current required for each electrode to produce an audible sound, using CT scans or comparable techniques [7,77]. A more uniform current distribution along the electrode array is thought to decrease pitch spread and thus increase sound quality, although there are mixed results on whether reducing pitch spread increases music perception scores [78]. This discovery challenges the long-held “one-size-fits-all” programming paradigm and suggests a future where CI users may have distinct, user-selectable “maps” optimized for different listening goals, such as conversation versus music enjoyment [76].

#### 5.1.2. Recapturing Temporal Fine Structure (TFS)

To address the loss of TFS, which is critical for low-frequency pitch perception, some strategies aim to explicitly encode this information. The most prominent examples are MED-EL’s Fine Structure Processing (FSP) strategy and its derivatives, FS4 and FS4-p [10]. These strategies operate on a hybrid principle: for mid-to-high frequency channels, they use a conventional envelope-based approach (like CIS), but for the three/four most apical channels, which correspond to the low-frequency region of the cochlea, they attempt to encode TFS by timing the stimulation pulses to the zero-crossings of the filtered waveform [10]. The FS4-p variant allows for parallel stimulation on these apical channels to potentially improve TFS representation further [10]. While many users report a subjective preference for FSP-type strategies, especially for music, objective performance data are mixed. Studies often show no significant advantage for speech perception in noise over modern envelope-based strategies, and benefits for music perception are not consistently demonstrated across all users [10,55,79]. This suggests that effectively delivering TFS information via an electrode array to a potentially degraded auditory nerve remains a significant bioengineering challenge.

### 5.2. The Role of Targeted Auditory Rehabilitation

Technological advancements alone may not be sufficient; the brain must learn to interpret the novel and often ambiguous electrical signal provided by the CI. Targeted auditory training aims to facilitate this process of experience-based neural plasticity, teaching the brain to extract more meaningful information from the degraded signal [80].

#### 5.2.1. From Listener to Participant: The Superiority of Auditory–Motor Training

A key principle emerging from rehabilitation research is that active engagement drives neural plasticity more effectively than passive listening [79]. This is demonstrated in a study by Chari et al. (2020) [72], which compared three groups of CI users over a one-month training period. The auditory–motor group, which used the “Contours” software to listen to and then reproduce melodic patterns on a keyboard, showed significantly greater improvement in melodic contour identification than both an auditory-only group (using the “AngelSound” listening software) and a no-training control group [73]. This finding provides strong evidence for the benefit of creating an “action-perception link,” where the motor act of producing a sound reinforces and refines the auditory system’s ability to perceive it [73]. This suggests that rehabilitative strategies that involve singing or playing an instrument may be particularly beneficial, as performing music engages and integrates multiple sensory and motor systems [81].

#### 5.2.2. Curated Listening: Computer-Based Music Training

A substantial body of research has demonstrated the efficacy of structured, computer-based music training programs (CBAT) for improving specific perceptual skills in CI users [80,82]. These programs have been shown to produce modest but statistically significant improvements in pitch discrimination, melody recognition, and timbre identification [80]. Training approaches can be broadly categorized as analytic (bottom-up), focusing on discriminating isolated acoustic features (e.g., telling if one tone is higher or lower than another), or synthetic (top-down), using more complex, real-world musical excerpts and leveraging cognitive skills like attention and context [74,83]. Both approaches have demonstrated benefits, suggesting that a comprehensive rehabilitation program should likely incorporate elements of both, starting with fundamental discrimination and progressing to more ecologically valid listening tasks [83]. Notably, evidence suggests that training one specific musical skill can produce a beneficial transfer effect to fundamental speech perception tasks. For example, Lo, McMahon, Looi, & Thompson (2015) demonstrated that melodic contour training, which focuses on identifying changes in pitch direction, has been shown to improve speech perception outcomes for CI recipients, specifically enhancing consonant discrimination in quiet and the perception of question/statement prosody [84]. These transfer effects are vital, demonstrating that music training is not just about musical enjoyment but is a powerful tool for improving core auditory processing that underlies social communication. Gfeller and colleagues (2002) conducted a 12-week self-administered CBAT program with 24 adult CI users, focusing on pitch, timbre, and melody [75]. The training resulted in a significant increase in instrument identification scores by approximately 20 percentage points and higher appraisal ratings [75]. Similarly, Galvin et al. (2007) found that CBAT focused on melodic contour identification led to significant performance improvements in 11 adult CI users [76]. More recently, Torppa and colleagues (2019) showed that pediatric CI users who participated in music and dance activities over 16 months performed similarly to their normal-hearing peers on pitch discrimination and word stress perception, demonstrating a direct transfer of musical training to a prosodic speech feature [80].

#### 5.2.3. Finding a Voice: Vocal Therapies and Singing Instruction

While most formal training studies have focused on perception, there is growing interest in therapies that directly target vocal production. Acoustic analyses of singing by prelingually deaf CI users reveal significant deficits in pitch accuracy but also a very wide range of individual performance. This variability suggests that, despite the baseline challenge of an altered auditory feedback loop, some individuals can learn to improve their vocal control through sustained practice. The experiences of “star” pediatric CI users who participate in formal music lessons and ensembles for many years show that exceptional levels of musical proficiency are attainable, reinforcing the potential of long-term, intensive vocal training [59].

Another important aspect of vocal therapies is individualized care, of which music therapy has been found to be relatively effective. A crucial part of music therapy is the ability of therapists to analyze progress and deliver appropriate, time-sensitive feedback to the patient, even altering the progress of the course if necessary [66,85,86]. More research is necessary to examine the breadth of the impact that personalized music therapy has on the rehabilitation process of CI users.

#### 5.2.4. The Unique Benefit of Group Singing and Choirs

While individualized auditory training and singing instruction are essential, the profound potential of structured, long-term group musical engagement (such as participating in a choir) merits specific attention, particularly in the pediatric population. The success of cochlear implantation is often measured in terms of individual perceptual gains, yet the act of singing in a choir introduces powerful psychosocial and motor-learning dynamics that can transcend the limitations of the degraded auditory signal.

A seminal study by Yang et al. (2019) demonstrated this potential by analyzing the singing proficiency of a choir comprising 10 prelingually deafened children with cochlear implants (mean age 9.5 years) who had received 21 months of formal music training [87]. The choir members with CIs achieved high accuracy in both pitch and tempo measures, performing on par with a control group of children with typical hearing. This exceptional outcome was attributed, in part, to an early start of music training following implantation and, critically, the use of bimodal hearing, which provided crucial low-frequency acoustic information for pitch perception [87].

This finding is paramount as it offers a powerful real-world counterpoint to the typical laboratory-based findings of pervasive pitch inaccuracy. It suggests that rigorous, sustained training, especially when anchored by the acoustic information provided by a residual-hearing ear, can effectively compensate for the functionally “broken” auditory–motor feedback loop. The group environment likely adds crucial, non-auditory scaffolding; singing alongside peers provides crucial visual, rhythmic, and potentially tactile cues, enhancing the social context and motivation necessary for long-term practice. This evidence reframes the singing deficit not as an absolute neurophysiological barrier, but as a challenge that can be overcome through intensive, multimodal, and socially scaffolded motor learning that shifts reliance from immediate acoustic feedback to stable, predictive motor planning.

### 5.3. Neuroplasticity and Individual Patient Factors

The success of any technology or therapy is ultimately mediated by the brain’s ability to adapt. Several biological and individual factors play a crucial role in determining a CI user’s potential for improvement.

#### 5.3.1. The Sensitive Period for Sound

The concept of a “sensitive period” in neurodevelopment is critical in pediatric cochlear implantation. During this window, the central auditory pathways exhibit maximal plasticity, making them highly receptive to stimulation-driven development [73]. Research by Sharma and colleagues (2009), using the P1 Cortical Auditory Evoked Potential (CAEP) as a biomarker for cortical maturation, has provided compelling evidence for this phenomenon [73]. Their work suggests that the sensitive period for optimal auditory development closes around the age of 7, with the best outcomes observed in children implanted before the age of 3.5 [73]. Implantation after this period is associated with abnormal cortical responses and evidence of cross-modal reorganization, where auditory areas of the brain may be recruited for other sensory functions, such as vision [73]. This cortical de-coupling, a functional disconnection between primary and higher-order auditory cortex, is thought to be a key mechanism underlying the end of the sensitive period [73]. This provides a powerful neuroscientific rationale for early implantation to maximize the brain’s innate capacity for auditory learning.

While this developmental sensitive period is critical for children, the adult brain also retains a remarkable capacity for plastic change following cochlear implantation. For adults with acquired hearing loss, the central auditory system has matured with normal acoustic input and must adapt first to hearing loss and then again to the novel electrical signal from the CI [88]. This process of adaptation can take months or years and is influenced by neurocognitive factors, but evidence suggests that active, engaged auditory training can help drive this experience-dependent plasticity, improving outcomes even in the mature brain [89].

#### 5.3.2. The Bimodal Advantage

For individuals with usable residual hearing in the non-implanted ear, a bimodal fitting (a CI in one ear and a hearing aid in the other) can offer a significant advantage for music perception [61,65]. The hearing aid delivers low-frequency acoustic information that is rich in TFS cues, which are largely absent from the CI signal [61,65]. This acoustic input provides crucial information for pitch perception, leading to significantly better melody recognition and greater music enjoyment compared to listening with the CI alone [61,65]. Interestingly, however, this clear perceptual benefit does not appear to automatically translate to superior production abilities. Studies that have acoustically analyzed singing have found no significant difference in pitch accuracy at a group level between bimodal users and bilateral CI users, suggesting that the relationship between perception and production is complex and that production skills may require specific training to develop [60].

However, this group-level finding masks a critical nuance. Within the bimodal group, the degree of residual hearing in the non-implanted ear is a significant predictor of success; one key study found that better unaided pure-tone thresholds were significantly correlated with more accurate singing (i.e., lower mean note deviation) [60]. This suggests that while the potential for better vocal production exists for bimodal users, it is highly dependent on the quality of the acoustic information they receive. The relationship between perception and production is therefore complex, and for many bimodal users, specific training may be required to translate their perceptual advantages into improved production skills.

#### 5.3.3. Psychosocial Scaffolding

Finally, it is crucial to recognize that successful auditory rehabilitation is not merely a clinical or technological process but a deeply human one, influenced by psychosocial factors. Qualitative research on highly successful “music-centric” pediatric CI users has illuminated the critical role of non-auditory factors in sustaining the long-term, intensive engagement required for exceptional musical achievement [60]. Intrinsic factors such as strong motivation, self-efficacy (the belief in one’s ability to succeed), a positive attitude toward challenges, and the integration of music into one’s personal identity are paramount [60].

It is also important to note that musical ability, including skills like pitch discrimination and singing in tune, has a wide and diverse range among those with typical hearing as well. This variation is not easily explained by formal psychacoustic measures alone, and it demonstrates that even in the absence of a hearing impairment, musical skills are often refined with practice, effort, and training over time, mirroring the developmental trajectory observed in CI users. This common need for training to realize musical potential links the rehabilitation process for CI users to the broader human experience of musical development.

These intrinsic factors are fundamentally supported by an external scaffold of extrinsic factors, most notably the family unit. Research by Looi, Torppa, Prvan, & Vickers (2019) comparing children with hearing loss to children with typical hearing found that parental attitudes regarding the importance of music in the family were similar across both groups [90]. Furthermore, parents of children with hearing loss generally do not view hearing loss as a contraindication to musical participation and actively encourage engagement and enjoyment, with children showing similar levels of music engagement and enjoyment regardless of their hearing status [90]. These along with extrinsic factors (encouraging parents who provide access to musical opportunities, inspiring and patient teachers, and the powerful social motivation and connection that comes from making music with peers in bands and orchestras) create an enabling environment [60,90]. This continuous family-based scaffolding, driven by similar levels of music engagement and enjoyment regardless of the child’s hearing status, is essential for transforming the potential afforded by the CI into realized musical and developmental outcomes.

This evidence reframes rehabilitation as a socio-ecological process, where success depends not only on the quality of the signal or the training software but on the rich network of human support that surrounds the listener. The most advanced technology can only create potential; it is this combination of targeted training, neural capacity, and psychosocial fuel that allows a CI user to realize that potential.

## 6. An Integrated Model of Musicality Post-Implantation

Synthesizing the extensive body of research on perception, production, and rehabilitation reveals a complex and multifaceted picture of musicality after cochlear implantation. The challenges are not merely a matter of a degraded signal but involve a cascade of consequences affecting the auditory–motor feedback loop the link between perception and production, and the very nature of musical enjoyment. A comprehensive understanding requires moving beyond isolated deficits to an integrated model that accounts for these interconnected factors.

### 6.1. The Relationship Between Perception and Production

The connection between auditory perception and vocal production in CI users is not straightforward. While it is intuitive to assume that one must be able to hear a distinction to produce it, the evidence suggests a more nuanced relationship that may vary depending on the specific auditory task.

In the domain of emotional prosody, a clear link between perception and production does appear to exist. A recent study by Chatterjee et al. (2023) found that in prelingually deaf children with CIs, their perceptual accuracy in identifying emotions was significantly predicted by the acoustic characteristics of their own vocal productions [91]. Specifically, children who produced larger acoustic contrasts between “happy” and “sad” sentences, in terms of both F_0_ variance (pitch modulation) and duration, were also better at identifying emotions in the speech of others [91]. This supports the hypothesis that perceptual access to the relevant acoustic cues is associated with, and perhaps a prerequisite for, the ability to modulate those same cues in one’s own voice. This finding is corroborated by Van de Velde et al. (2019), who also observed a weak but significant correlation between emotion perception and production abilities in their cohort of pediatric CI users [68].

However, this direct link does not seem to hold universally across all aspects of prosody. Research on the production of linguistic prosody, such as placing focal stress on a word in a sentence, complicates the picture. Van de Velde et al. (2019) found that CI children were capable of producing different types of focal accents appropriately, yet they were unable to perceptually discriminate between those same accent types when listening [68]. This intriguing dissociation suggests that some production skills may be learned through alternative pathways, perhaps relying on more holistic motor pattern learning, imitation of durational and intensity cues, or feedback from communication partners, rather than being solely dependent on fine-grained auditory perceptual accuracy. The nature of this perception-production link is likely shaped by developmental timing; for instance, the integration of auditory and motor systems for vocal control may be most malleable during an early sensitive period, and the deficits seen in CI users may reflect the consequences of absent or degraded auditory input during this critical window. In post-lingually deafened adults, this integration must occur within a mature system that may have already undergone reorganization due to hearing loss, presenting a different set of challenges for rehabilitation [88].

### 6.2. The Disconnect Between Perception and Appraisal

One of the most critical and counterintuitive findings in the field is the apparent disconnect between objective music perception skills and the subjective experience of musical enjoyment. The research of Wright & Uchanski (2012) is pivotal in this area [9]. In a comprehensive study, they administered a battery of music perception tests (assessing melody, timbre, rhythm, etc.) and also asked CI users to provide appraisal ratings (i.e., pleasantness or likability) for various musical excerpts [9]. Their analysis revealed no statistically significant correlation between the participants’ perceptual scores and their appraisal ratings.

This “perception-appraisal gap” has profound implications for how “success” in music rehabilitation is defined and measured. It demonstrates that improving a user’s ability to, for example, identify a melodic contour does not guarantee that they will find music more enjoyable. Musical enjoyment is a complex, holistic experience that transcends the sum of its acoustic parts. It is deeply influenced by factors that are often well-preserved in CI users, such as rhythm, lyrical content, memory, social context, and cultural significance [6]. Many CI users derive considerable pleasure from music by focusing on its strong rhythmic elements or by following the lyrics of a familiar song, which can trigger a rich mental representation of the music from their hearing memory, even if the real-time melodic information is distorted [6].

The relationship between perception and musical outcomes is further complicated by the ongoing nature of auditory development and the varied impact of training. While core pitch perception (e.g., discrimination thresholds) often shows limited short-term improvement, studies on children with sensorineural hearing loss (including CI users and hearing aid users) suggest that music training can yield significant benefits in foundational listening skills. For example, Lo, Looi, Thompson, & McMahon (2020) demonstrated that a modest amount of music training significantly improved perception of speech-in-noise, question/statement prosody, musical timbre, and spectral resolution in children [92]. Importantly, although this training did not lead to immediate benefits in emotional prosody or pure pitch perception, the demonstrated improvements in spectral resolution suggest that training first enhances the fidelity of the auditory input [92]. This supports the hypothesis that long-term musical engagement facilitates neuroplasticity, potentially allowing foundational auditory processing skills to mature and setting the stage for later, long-term improvements in complex pitch tasks that are currently masked by the constraints of the CI signal. This suggests that clinical and research efforts must address two distinct goals: enhancing perceptual accuracy and improving subjective quality of life, as progress in one domain does not automatically confer benefit in the other.

### 6.3. A Holistic Framework for Auditory Rehabilitation

The collective evidence points to the need for a paradigm shift in the clinical management of CI users, moving from a narrow, speech-centric model focused on audiometric outcomes to a comprehensive, patient-centered framework that addresses the whole person. The finding that traditional speech recognition scores are somewhat inaccurate predictors of overall QoL serves as a powerful catalyst for this change [25]. A holistic model must incorporate measures and interventions that target real-world functioning and well-being.

The World Health Organization’s International Classification of Functioning, Disability, and Health (ICF) provides an excellent theoretical model for such an approach [9,93]. The ICF framework conceptualizes health not just as the absence of impairment but as a dynamic interaction between a health condition, body functions (e.g., sound detection), activities (e.g., listening, conversation), participation (e.g., family relationships, employment), and contextual (environmental and personal) factors [9]. In parallel, specialized patient-reported outcome measures have been developed to capture this broader view of success. Instruments like the Nijmegen Cochlear Implant Questionnaire (NCIQ) and the Cochlear Implant Quality of Life (CIQOL) suite assess domains far beyond sound perception, including social interaction, self-esteem, emotional well-being, and listening effort, providing a more complete picture of the patient’s lived experience [94,95]. However, given the significant impact of music and singing on a user’s social engagement and identity, researchers have developed even more targeted measures. The Music-Related Quality of Life (MuRQoL) questionnaire, pioneered by Dritsakis and colleagues, specifically addresses how music experiences, abilities, attitudes, and activities impact QoL [96]. The MuRQoL defines quality of life as a function of music experiences and their importance, capturing self-reported music perception and engagement, and is uniquely suited to guide and evaluate music aural rehabilitation programs [96]. By incorporating such specialized metrics, clinicians can capture the nuanced psychosocial factors overlooked by traditional audiometric testing.

This conceptual shift is being translated into practice through integrated care models. For example, manufacturer-led initiatives like the Cochlear Family program provide a support network that connects new users with experienced mentors and offers resources for rehabilitation and device use [80,97]. Such programs exemplify a holistic approach by combining traditional sensory management with psychosocial counseling and peer support, acknowledging that successful adaptation to a CI is a journey that involves not only learning to hear but also navigating the emotional and social challenges of hearing loss [80,97]. By treating the whole person and not only targeting hearing abilities, such programs aim to maximize how they can thrive in their daily lives [97].

## 7. Conclusions and Future Directions

### 7.1. Summary of Key Findings

The journey of a CI user into the world of complex sounds is one of remarkable gains and persistent challenges. This review has synthesized evidence demonstrating that while CIs are exceptionally successful at restoring speech perception, their fundamental design creates a spectrally degraded signal that profoundly disrupts the perception of pitch-based information. This core limitation cascades into significant deficits in music perception and the interpretation of vocal prosody. In turn, the impoverished auditory signal breaks the auditory–motor feedback loop, leading to characteristic inaccuracies in vocal singing, particularly in pitch control.

Pathways to improvement are necessarily multifaceted, requiring a synergistic combination of technological innovation (e.g., current steering, TFS processing, image-guided programming), targeted, brain-based rehabilitation that emphasizes active, auditory–motor engagement, and a deep appreciation for the individual factors that govern success. These include the brain’s capacity for neuroplasticity, which is maximal during the sensitive developmental period in children but remains a critical factor for adaptation throughout adulthood, and the crucial role of psychosocial support.

### 7.2. Future Directions

While significant progress has been made, critical gaps in our knowledge remain. A forward-looking research agenda must acknowledge the high degree of individual variability in CI performance, necessitating a greater focus on key patient-related and psychosocial confounding variables.

First, cognitive factors, such as auditory working memory and cognitive control, are common deficits in hearing-impaired adults and are tied to reduced quality of life and difficulty enjoying music and social listening [83]. Future research should explicitly separate the influence of cognitive function (e.g., task-switching) from auditory input quality, using cognitive batteries to predict success in both music perception and production training outcomes.

Second, musical and experiential factors are crucial, as highly successful pediatric CI users demonstrate that strong intrinsic motivation, self-efficacy, and social scaffolding (encouraging parents, inspiring teachers) are critical for realizing exceptional musical proficiency [89]. Research should investigate the optimal timing and intensity of auditory–motor training (e.g., active singing lessons) and develop standardized tools to measure motivation and self-efficacy as predictors of long-term singing accuracy.

Third, socioeconomic status (SES) represents a powerful systemic barrier; individuals from lower income quintiles are more likely to qualify for a CI but less likely to pursue implantation [98]. Furthermore, while household income may not strictly predict the use of computer-based auditory training post-activation, access to technology (smartphone/computer) is highly predictive of participation [99]. Future research must therefore explore the downstream impact of SES barriers on music-related outcomes, specifically correlating income/geographic access with long-term engagement in, and the effectiveness of, specialized music therapy and auditory–motor training programs.

Beyond these individual factors, there is a pressing need for well-controlled, longitudinal research that moves beyond the short-term training studies that currently dominate the field. Tracking the developmental trajectory of music and prosody skills over years, not just weeks, is essential for understanding the true impact of sustained musical engagement and the long-term evolution of experience-based plasticity, particularly in pediatric cohorts [83,95]. Second, to compare findings across studies and better reflect real-world benefit, the field must move toward the development and adoption of standardized and ecologically valid outcome measures. This includes not only perceptual tests, such as the MuSIC test battery, but also validated measures of sound quality and, crucially, patient-reported outcome measures like the CIQOL suite that capture the user’s lived experience [95]. Furthermore, research should continue to push the boundaries of technology beyond incremental improvements to current sound processing. Promising areas include investigating AI-driven music pre-processing to simplify musical recordings in real-time and exploring multimodal stimulation, such as electro-haptic feedback, to supplement the impoverished auditory signal. Specifically, future work should aim to delineate the boundaries and mechanisms of the sensitive periods for both music and singing acquisition in the CI population, leveraging modern brain imaging techniques to track developmental changes in auditory–motor pathways. Finally, the future of CI rehabilitation lies in a personalized, predictive approach. This requires leveraging neuroimaging and electrophysiology to identify presurgical neural markers that can predict which patients are likely to struggle with complex sounds, allowing for the triaging of patients into tailored rehabilitation pathways from the outset [95,100].

### 7.3. Challenges of Implementation and Access

While research validates the benefits of advanced programming and specialized music rehabilitation, the successful translation of these findings into clinical practice is hindered by significant real-world barriers. The primary challenges to widespread implementation are rooted in access, cost, and clinician awareness.

Firstly, despite the profound benefits of cochlear implantation, the rate of CI utilization in countries like the United States remains low (around 6% for adults and 50% for children who could benefit), lagging behind other developed nations [101]. This underprovision is often compounded by financial issues, including the cost of insurance and accessibility barriers due to restrictive state Medicaid policies. These financial and geographic barriers prevent many eligible patients from even receiving the implant, let alone the specialized post-implant rehabilitation that addresses music and voice perception. Secondly, implementation is hampered by a lack of specialized referral pathways and low awareness of the benefits of CIs and specialized rehabilitation among general healthcare professionals, including primary care physicians and audiologists who do not specialize in CIs [102]. This knowledge gap means that opportunities for intervention, particularly during the sensitive developmental period in children, are often missed [102]. Thirdly, implementing sophisticated, personalized rehabilitation like Auditory–Motor Training or music therapy requires significant investment in clinician training and resources, which may not be universally available in rural areas.

Overcoming these barriers requires a shift toward a comprehensive public health approach. This includes proactive identification of candidates, public health outreach programs targeting schools and rural areas, and advocacy to ensure financial policies (such as state Medicaid rules) are aligned with national clinical guidelines for CI provision and rehabilitation access.

### 7.4. Clinical Implications and Recommendations

The insights gained from this body of research should catalyze a shift in standard clinical practice, bridging the gap between the laboratory and the clinic. A primary recommendation is the adoption of a holistic care model, moving beyond a framework that centers exclusively on device programming and speech testing. Incorporating holistic frameworks, such as the ICF, and integrating psychosocial support, self-efficacy training, and patient-centered goal setting are essential for addressing the full scope of a patient’s needs [80,97]. Within this model, music and prosody should be incorporated into standard rehabilitation. Music-based auditory training and exercises focused on perceiving and producing emotional and linguistic prosody should become an accessible component of aural rehabilitation for all CI users, as these are foundational skills that impact daily communication and well-being, not just niche interests.

Ultimately, clinical practice must prioritize quality of life. As the goal of cochlear implantation is to improve a person’s life, validated quality of life measures, such as the CIQoL instruments, should be routinely incorporated as a primary outcome metric [95]. Notably, when music or singing is identified as a patient goal, clinicians should integrate music-specific quality of life tools, such as the MuRQoL questionnaire [96]. This combined, dual-metric approach ensures that interventions are evaluated based on their comprehensive real-world impact, shifting the clinical focus from what a patient can hear in a booth to how that patient lives, feels, and connects in the world. By embracing this broader vision, the field can move closer to restoring not just hearing, but the rich and varied soundscape of human experience.

## Figures and Tables

**Table 1:** Summary of Perceptual Deficits and Their Consequences Among Cochlear Implant Users.

Auditory Domain	Specific Perceptual Deficit	Primary CI Limitation(s)	Real-World Consequence
Pitch & Melody [23]	Poor melody recognition; difficulty discriminating small pitch changes.	Poor spectral resolution; loss of Temporal Fine Structure (TFS).	Music sounds “out of tune”; difficulty recognizing familiar songs without lyrics.
Timbre [24]	Difficulty distinguishing between different musical instruments or singers’ voices.	Coarse spectral resolution; channel interaction (“spectral smearing”).	Instruments sound muddled or similar; difficulty appreciating orchestral texture.
Vocal Prosody [25]	Impaired recognition of vocal emotion, sarcasm, and pragmatic intent.	Poor pitch perception due to degraded spectral cues and loss of TFS.	Frequent misunderstandings in conversation; reduced social connection and empathy.
Speech-in-Noise [26]	Significantly reduced ability to understand speech in the presence of background noise.	All of the above; inability to use fine structure cues to separate sound sources.	Difficulty in restaurants, group conversations, and other complex listening environments.

**Table 2:** Critical Appraisal of Key Quantitative Studies on Auditory–Motor Function and Singing in Cochlear Implant (CI) Users.

Authors	Design/Methodology	Population	Key Quantifiable Result (CI Users)	Limitations/Nuances
Gautam et al., (2020) [59]	Real-time sensorimotor adaptation paradigm using vocal pitch perturbation.	Adult CI recipients.	Compensatory vocal response initiated only at 6 semitone feedback shifts.	Small, specialized cohort; measures compensatory response threshold, not everyday pitch accuracy.
Xu et al., (2009, 2022) [60,65]	Acoustic analysis of sung melodies; correlation analysis of residual hearing.	Pediatric CI users (prelingually deafened), including bimodal and bilateral users.	Mean note deviation: 2.3 to 2.7 semitones; Melodic contour accuracy: 52.3%. Bimodal advantage correlated to residual hearing (r = 0.582).	Focus on prelingually deaf children, whose developmental trajectories differ from adults. Wide variability in individual performance.
Gfeller et al., (2007) [22]	Auditory discrimination tasks (pure-tone frequency difference limens).	Adult CI users.	Pitch discrimination threshold: ~3 semitones.	Measures perception of isolated tones in a controlled setting, which may overestimate ability to process complex pitch in real music.
Chari et al., (2020) [72]	Controlled training study comparing Auditory–Motor vs. Auditory-Only training.	Adult CI users.	Auditory–motor training provided significantly greater improvement in melodic contour recognition.	Short training duration (1 month); findings relate to perception (recognition) improvement, not directly to sustained production (singing) accuracy.
Sharma et al., (2009) [73]	P1 Cortical Auditory Evoked Potential (CAEP) biomarker measurement.	Pediatric CI Users.	Sensitive period closes around age 7; best outcomes when implanted before 3.5 years.	CAEP is a neurophysiological biomarker; correlation with behavioral singing outcome requires further long-term study.
Wright & Uchanski (2012) [9]	Objective perception tests (melody, timbre, rhythm) alongside subjective appraisal ratings.	Adult CI users and simulated CI listeners.	Objective scores did not correlate statistically with subjective appraisal ratings (Perception-Appraisal Gap).	Relies on subjective, self-reported appraisal ratings; inclusion of CI simulators may not perfectly mirror electric hearing.

## Data Availability

No new data were created or analyzed in this study.

## Abbreviations

The following abbreviations are used in this manuscript:

CI	Cochlear Implant
CIS	Continuous Interleaved Sampling
ACE	Advanced Combination Encoder
NH	Normal Hearing
TFS	Temporal Fine Structure
MBEA	Montreal Battery of Evaluation of Amusia
CAMP	Clinical Assessment of Music Perception
FSP	Fine Structure Processing
CBAT	Computer-Based Music Training
CAEP	Cortical Auditory Evoked Potential
ICF	International Classification of Functioning
NCIQ	Nijmegen Cochlear Implant Questionnaire
CIQOL	Cochlear Implant Quality of Life
